# Accelerometer-Measured Physical Activity Data Sets (Global Physical Activity Data Set Catalogue) That Include Markers of Cardiometabolic Health: Systematic Scoping Review

**DOI:** 10.2196/45599

**Published:** 2023-07-19

**Authors:** Jonah J C Thomas, Amanda J Daley, Dale W Esliger, Victoria E Kettle, April Coombe, Emmanuel Stamatakis, James P Sanders

**Affiliations:** 1 School of Sport, Exercise and Health Science Loughborough University Loughborough United Kingdom; 2 National Centre for Sport and Exercise Medicine Loughborough University Loughborough United Kingdom; 3 Centre for Lifestyle Medicine and Behaviour Loughborough University Loughborough United Kingdom; 4 Lifestyle, National Institute of Health Research Leicester Biomedical Research Centre Leicester United Kingdom; 5 Public Health, Epidemiology and Biostatistics Institute of Applied Health Research University of Birmingham Birmingham United Kingdom; 6 Charles Perkin Centre Faculty of Medicine and Health Science University of Sydney Sydney Australia

**Keywords:** sedentary behavior, device measured, data harmonization, open science, big data

## Abstract

**Background:**

Cardiovascular disease accounts for 17.9 million deaths globally each year. Many research study data sets have been collected to answer questions regarding the relationship between cardiometabolic health and accelerometer-measured physical activity. This scoping review aimed to map the available data sets that have collected accelerometer-measured physical activity and cardiometabolic health markers. These data were then used to inform the development of a publicly available resource, the Global Physical Activity Data set (GPAD) catalogue.

**Objective:**

This review aimed to systematically identify data sets that have measured physical activity using accelerometers and cardiometabolic health markers using either an observational or interventional study design.

**Methods:**

Databases, trial registries, and gray literature (inception until February 2021; updated search from February 2021 to September 2022) were systematically searched to identify studies that analyzed data sets of physical activity and cardiometabolic health outcomes. To be eligible for inclusion, data sets must have measured physical activity using an accelerometric device in adults aged ≥18 years; a sample size >400 participants (unless recruited participants in a low- and middle-income country where a sample size threshold was reduced to 100); used an observational, longitudinal, or trial-based study design; and collected at least 1 cardiometabolic health marker (unless only body mass was measured). Two reviewers screened the search results to identify eligible studies, and from these, the unique names of each data set were recorded, and characteristics about each data set were extracted from several sources.

**Results:**

A total of 17,391 study reports were identified, and after screening, 319 were eligible, with 122 unique data sets in these study reports meeting the review inclusion criteria. Data sets were found in 49 countries across 5 continents, with the most developed in Europe (n=53) and the least in Africa and Oceania (n=4 and n=3, respectively). The most common accelerometric brand and device wear location was Actigraph and the waist, respectively. Height and body mass were the most frequently measured cardiometabolic health markers in the data sets (119/122, 97.5% data sets), followed by blood pressure (82/122, 67.2% data sets). The number of participants in the included data sets ranged from 103,712 to 120. Once the review processes had been completed, the GPAD catalogue was developed to house all the identified data sets.

**Conclusions:**

This review identified and mapped the contents of data sets from around the world that have collected potentially harmonizable accelerometer-measured physical activity and cardiometabolic health markers. The GPAD catalogue is a web-based open-source resource developed from the results of this review, which aims to facilitate the harmonization of data sets to produce evidence that will reduce the burden of disease from physical inactivity.

## Introduction

### Background

Regular moderate to vigorous intensity physical activity reduces the risk of cardiovascular disease and improves individuals’ cardiometabolic profile including markers such as waist circumference, high-density lipoprotein (HDL) cholesterol, and triglycerides [1,2]. Although self-reported measures of physical activity have been used extensively in previous research, technological advances have led to accelerometers becoming widely available, making them feasible to be used at scale within health research. Accelerometers are small devices that measure acceleration, and from this measurement, estimates of the intensity of physical activity can be derived. This has led to an increase in the number of cohort or health surveillance studies that have collected device-measured physical activity data alongside cardiometabolic health markers. One such data set is the National Health and Nutrition Examination Survey (NHANES), which collected accelerometer-measured physical activity as well as blood pressure, blood lipids, and blood glucose data from approximately 5000 adults [3]. Furthermore, NHANES provides the opportunity to answer new health research questions without the need for additional and potentially expensive data collection. Other notable large health surveillance data sets that measured physical activity using an accelerometer and collected cardiometabolic health markers include the Canadian Health Measures Survey 2007-2009 and 2009-2011 and the Health Survey for England 2008 [4]. Several large cohort studies have also introduced accelerometry measures, including the UK Biobank [5,6] and the 1970 British Cohort [7].

In recent years, efforts have been made to pool accelerometer-measured physical activity data sets alongside health-related markers. An example of a large-scale harmonization initiative is the International Children’s Accelerometer Database [8], which has pooled data from 20 studies that collected physical activity and health marker data in children. The International Children’s Accelerometer Database has advanced the field by enhancing our understanding of the correlation between children’s physical activity levels and health markers, enabling the examination of geographical and interstudy variances. There are several other notable studies that have used harmonization methodologies in adults [9,10]. Harmonized data sets can increase statistical power by generating larger sample sizes as well as increase the heterogeneity (eg, ethnicity, body mass, and body fat percentage) of the data, potentially enhancing the representation of the overall study sample.

A necessary first step to current harmonization efforts, after defining a research question, is the need to perform an initial review to identify all the data sets that may be available for inclusion. Furthermore, the need for this review process is resource intensive, requiring considerable time and effort to complete in a comprehensive manner, limiting the feasibility of such endeavors. Therefore, providing a shared resource to reduce this burden will provide benefits to the wider research community. In addition, to harmonize data sets effectively, a large amount of information about each variable collected must be retrieved, including the methodologies used [11]. The systematic methodological process of accelerometer harmonization is becoming increasingly important as the device used and the data analytic decisions taken impact the derived estimates of physical activity that are available [12,13]. This review aimed to identify previously collected data sets to ease the harmonization process but did not aim to perform or instruct on the data harmonization process.

### Objectives

Therefore, the aim of this scoping review was to identify and map the contents of the available data sets that have collected data on accelerometer-measured physical activity and cardiometabolic health markers.

## Methods

### Overview

To ensure that the methodology used was consistent with that used in previous scoping reviews, the framework constructed by Arksey and O’Malley [14] and later developed by Levac et al [15] was followed. The review was registered on the Open Science Framework [16] and was written in accordance with the PRISMA-ScR (Preferred Reporting Items for Systematic Reviews and Meta-Analyses extension for Scoping Reviews) checklist [17]. The methodology for this review followed 5 stages.

### Stage 1: Identify the Question

Three research aims were derived to focus on the review and achieve the overall aim:

1. To systematically identify data sets that have measured physical activity using accelerometers and cardiometabolic health markers using either an observational or interventional study design
2. To identify the key characteristics of eligible data sets (eg, study location, population of interest, and the device used to collect the physical activity data and cardiometabolic health markers that were simultaneously collected)
3. To determine the access status (open, upon request, or restricted) of eligible data sets to assess the feasibility of conducting future harmonized data analysis

### Stage 2: Search for Literature

The search strategy was developed by an information specialist (AC) who also completed the study searches between February 2, 2021, and February 10, 2021. The search keywords were determined in consultation with the research team. The search terms stemmed from 3 main categories: physical activity (physical*, activ*, and exercise*), accelerometer (acceleromet* and activity monitor*), and study design (cross-sectional and randomized). Searches were devised and tested using MEDLINE. The search strategy was then adapted for other databases, including Embase, CINAHL, CENTRAL, SportDiscus, OpenGrey, WHO ICTRP, ClinicalTrials.gov, and Conference Proceedings Citation Index. The full search strategy is provided in Multimedia Appendix 1. The searches were limited to human adults aged ≥18 years. No date restriction was applied to the searches. No language restriction was applied, with papers not written in English being translated using web-based software. A brief update search was performed in PubMed, covering the period from the initial search to the final analysis (September 1, 2022).

### Stage 3: Study Selection

The reports identified by the search were uploaded into Covidence (Veritus Health Initiative), and automatic deduplication was performed. Title and abstract screening of each report was independently performed by 2 researchers (JJCT and 1 of AJD, VEK, or JPS). Disagreements were resolved through discussion between the 2 researchers. All the study titles and abstracts were screened based on the inclusion and exclusion criteria outlined in Textbox 1. Although this review aimed to systematically identify eligible data sets in adults, if a data set had collected data on adults but also included participants aged <18 years, the data set would still be deemed eligible. Data sets collected from clinical populations (individuals living with hypertension or type 2 diabetes) were included, provided that the participants were free living.

**Inclusion and exclusion criteria.**

**Inclusion criteria**

- Observational, longitudinal, or trial-based studies of any design
- Adult participants (aged ≥18 years)
- Measures physical activity using an accelerometric device (for this study, an accelerometric device excludes mobile phones or commercially available activity trackers that contain an accelerometer) for at least 24 hours
- Published in any language (this is a criterion of the journal article or report, rather than the data set)
- A sample size of >400 participants (unless collected in low- and middle-income countries, as defined by the World Bank [18])
- Collected at least 1 cardiometabolic marker

**Exclusion criteria**

- Nonhuman populations
- Only reported sleep exposures

**Figure 1:**
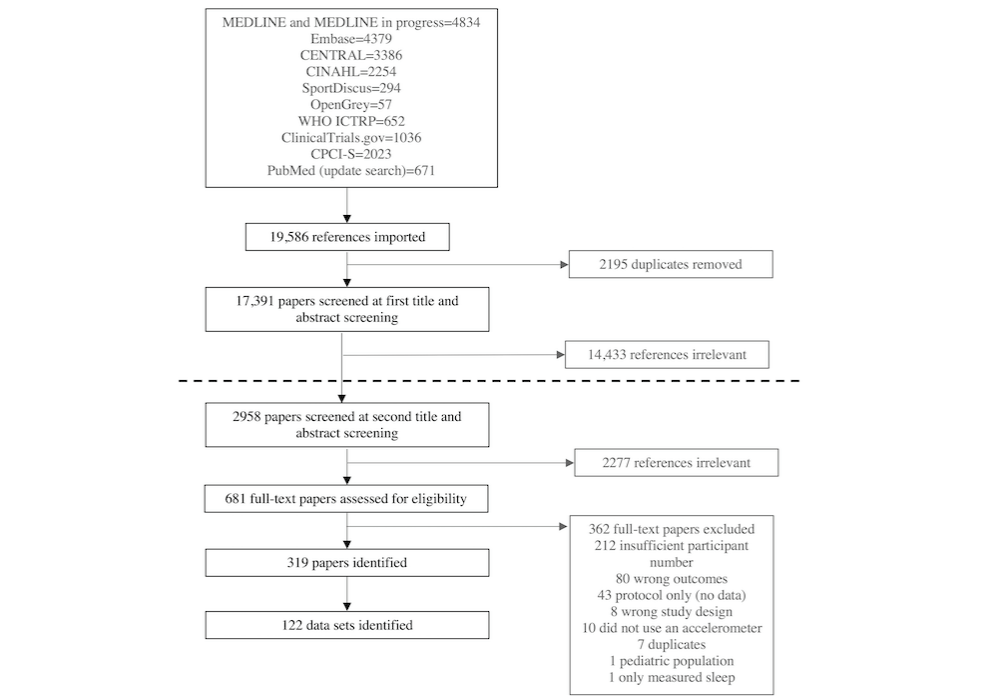
PRISMA (Preferred Reporting Items for Systematic Reviews and Meta-Analyses) diagram of the screening and extraction process. The black dotted line represents the separation between the first and second stage of screening. CPCI-S: Conference Proceedings Citation Index–Science; WHO ICTRP: World Health Organization International Clinical Trials Registry Platform.

After the title and abstract screening, the number of reports for full-text screening was deemed too large to be feasible (n=2195; Figure 1). Therefore, a second title and abstract screening step was performed with more specific inclusion and exclusion criteria (Textbox 1). A revised sample size inclusion criterion of at least 400 participants was applied, which was chosen to be consistent with previous studies [8,19]. To ensure that data sets from as many countries as possible were included, a reduced sample size criterion (>100 participants) for low- and middle-income countries (LMICs) as defined by the World Bank [18] was applied. This reduced participant sample size criterion was also applied because it was expected that data sets in LMICs would have smaller sample sizes than high-income countries, as typically there is less research funding available for the development of such data sets in LMICs. The desire to include data sets from LMICs (eg, African nations) is also important because these countries tend to have a greater ethnic diversity of citizens, and it is critical to ensure that there is data representation in this catalog from across the ethnicity spectrum. After the second screening process, a full-text screening was conducted by a single researcher (JJCT) for the remaining studies, and the reasons for exclusion were recorded.

### Stage 4: Describing and Charting the Data

Data extraction was performed by a single researcher (JJCT). Descriptive information about each data set was extracted from the eligible reports. To extract as much outcome information as possible about the data sets, several additional sources were used, including websites, methodology or protocol reports or publications that describe the data set cohort, and individual articles stemming from the data set. If the information was irretrievable at this stage, another researcher verified that it could not be retrieved (JPS), and the variable was marked as unknown.

Several variables were extracted regarding each data set: the number of participants, mean age, the proportion of male and female participants, country and continent of data collection, and the data access status. Information was extracted regarding the physical activity measurement method, including accelerometer brand and model, and deployment protocol (eg, the number of days the accelerometer was worn for and the raw sampling frequency [Hz] of the accelerometer data). Furthermore, the cardiometabolic health markers measured were recorded. In this review, cardiometabolic health markers were defined as follows: height, weight, waist circumference, hip circumference, fat mass, visceral fat, systolic blood pressure, diastolic blood pressure, resting heart rate, HDL cholesterol, low-density lipoprotein cholesterol, total cholesterol, triglycerides, very low–density lipoprotein, glycated hemoglobin, blood glucose, blood insulin, and oral glucose tolerance test.

Owing to the large number of health markers extracted, outcomes were combined into 4 categories: anthropometry (ie, height, body mass, and waist circumference), blood pressure, blood lipids (ie, HDL cholesterol), and blood glucose control (ie, blood glucose and glycated hemoglobin; outlined in Multimedia Appendix 2).

### Stage 5: Collating, Summarizing, and Reporting the Extracted Data

The retrieved information is presented in 2 ways. Following the example of the Maelstrom Catalogue, a web-based catalog was created to summarize the identified data sets. This catalog is available as a web-based data visualization tool [20] and will hereby be referred to as the Global Physical Activity Data set (GPAD) catalogue. The GPAD catalogue provides an overview of the data sets identified in this review and the markers assessed in each data set. Second, a narrative summary of the key findings was produced to highlight the patterns found within the identified data sets.

## Results

### Overview

The database searches yielded 19,586 references. After the duplicates were removed (n=2195), title and abstract screening was performed on 17,391 articles, and 2958 reports were identified as eligible for full-text review. The second-stage title and abstract screening with stricter exclusion criteria returned 556 papers for full-text screening. The updated search returned 671 reports (with no duplicates), from which an additional 109 papers were included for full-text screening, making the number of total papers that went through full-text screening to be 655. From these, 362 papers were excluded, resulting in the identification of 319 reports (Multimedia Appendix 3). From these, 122 data sets were identified (Multimedia Appendix 4). The full screening process is detailed in Figure 1, and all the identified data sets are included in Table 1.

**Table 1:** Characteristics of the data sets identified as part of this review.

Data set or study name	Study type	Country of data collection	Mean age (years)	Sample size, n	Device brand and model	Placement	Number of days of wearing the device
Framingham Heart Study Generation 3 [21]	Longitudinal	United States	40	4094	Phillips Actical	Hip	8
Framingham Heart Study Omni 2 [22]	Longitudinal	United States	—^a^	410	Phillips Actical	Hip	8
Malaysian Government Employees with MetS^b^ [23]	Observational	Malaysia	40	490	Kenz	Waist	7
EVIDENT^c^ [24]	Observational	Spain	54.92	636	Actigraph GT3x	Right waist	7
Generation 100 [25]	Interventional	Norway	72	1567	Sensewear Armband and Actigraph GT3x	Arm and waist	7
National Health and Nutrition Examination Survey 2003-2004^d^ [26]	Observational	United States	48	6830	Actigraph AM-7164	Waist	7
National Health and Nutrition Examination Survey 2005-2006^d^ [27]	Observational	United States	—	3081	Actigraph AM-7164	Waist	7
UK Biobank [28]	Observational	United Kingdom	57	103,712	Axivity AX3	Wrist	7
Tasmanian Older Adults Cohort [29]	Observational	Australia	66	636	Actigraph GT1M	—	7
Malaysian Government Employees [30]	Observational	Malaysia	32	233	Kenz	Waist	—
OPACH^e^ [31]	Observational	United States	—	7058	Actigraph GT3x	Waist	7
CARDIA^f^ year 20^d^ [32]	Longitudinal	United States	—	2332	Actigraph 7164	Waist	—
CARDIA year 30^d^ [32]	Longitudinal	United States	—	2332	Actigraph wGT3X-BT	Waist	—
Walking Away From Diabetes [33]	Interventional	United Kingdom	63	725	Actigraph GT3x	Waist	7
PROPEL^g^ [34]	Interventional	United Kingdom	59.4	1308	Actigraph GT3X	Waist	7
HCHS^h^ or SOL^i^ 2008-2011 [35]	Longitudinal	United States	—	8049	Actical B1	Waist	7
HCHS/SOL 2014-2017 [35]	Longitudinal	United States	—	8049	Actical B1	Waist	7
Dallas Heart Study 2 [36]	Longitudinal	United States	—	3401	Actical	—	—
EpiFloripa Aging Cohort [37]	Observational	Brazil	73.9	604	Actigraph GT3x and GT3x+	Right hip	7
Japanese metabolic syndrome [38]	Observational	Japan	47	483	Omron HJA-350IT	Right hip	7
Association of metabolic syndrome and blood pressure nondipping profile in untreated hypertension [39]	Observational	Spain	48.7	1770	Ambulatory Monitoring Mini-motion logger	Wrist	—
LIFE [40]	Interventional	United States	—	1635	Actigraph GT3X	Hip	7
Health 2011 Study [41]	Observational	Finland	53	1398	Hookie AM20	Hip	7
PATH^j^ [42]	Interventional	United States	51	434	Mini-Mitter Actical	—	7
Health Survey for England [5]	Observational	United Kingdom	51	2131	Actigraph GT1M	Waist	7
ADDITION-PRO [43]	Observational	Denmark	58.5	2082	Actiheart	Chest	7
Healthy Aging Initiative [44]	Observational	Sweden	70.5	3343	Actigraph GT3x+	Hip	7
Feedback, Awareness and Behavior study [45]	Observational	United Kingdom	47	453	Actiheart	—	6
Being physically active modifies the detrimental effect of sedentary behavior on obesity and cardiometabolic markers in adults [46]	Observational	Chile	—	314	Actigraph GT1M	Hip	7
Body composition among elderly and its relationship with physical activity pattern [47]	Observational	Iran	—	368	Actigraph	—	—
Maastricht Study [48]	Observational	Netherlands	59.7	10,000	ActivPAL 3	Thigh	7
Copenhagen City Heart Study [49]	Longitudinal	Denmark	—	1053	Actigraph GT3x	Thigh and waist	7
ACTION! Worksite Wellness Program [50]	Observational	United States	—	850	Actigraph 7164	—	7
Nutrition and Exercise Intervention Study [51]	Interventional	Japan	—	1085	ActiMarker	Thigh	14
ALSPAC^k^ Mother Cohort [52]	Longitudinal	United Kingdom	—	4834	Actigraph 7164	—	—
Combined effects of obesity and objectively measured daily physical activity on the risk of hypertension in middle-aged Japanese men: a 4-year prospective cohort study [53]	Observational	Japan	49	426	Kenz Lifecorder	—	7
PREVIEW^l^ [54]	Interventional	Denmark, Finland, Netherlands, United Kingdom, Spain, Bulgaria, Australia, and New Zealand	—	2500	Actigraph ActiSleep+	Waist	7
CHMS^m^ 2007-2009 [55]	Observational	Canada	—	2832	Actical	Waist	7
CHMS 2009-2011 [55]	Observational	Canada	—	2103	Actical	Waist	7
North Finland Birth Cohort 1966 [56]	Longitudinal	Finland	46.6	3443	Polar Active	Nondominant wrist	14
Food4Me [57]	Interventional	Ireland, Netherlands, Spain, Greece, United Kingdom, Poland, and Germany	40.1	1441	Phillips DirectLife	Pocket, belt, necklace, or bra	180 days
DPHACTO^n^ [58]	Observational	Denmark	45.1	669	Actigraph GT3x	Thigh	7
Abstract P022: determinants of energy balance: differences related to body weight and body composition [59]	Abstract only	—	—	430	SenseWear	—	—
Device-based measures of sedentary time and physical activity are associated with physical fitness and body fat content [60]	Observational	Finland	26	415	Hookie AM20	Waist	7
MAPEC^o^ [61]	Interventional	Spain	55.6	2156	Mini-motion logger	Wrist	—
Al-Andulus [62]	Observational	Spain	49.9	653	Actigraph GT3X+	Lower back	9
British Regional Heart Study [63]	Longitudinal	United Kingdom	78.4	1566	Actigraph GT3x	Right hip	7
Pelotas [64]	Longitudinal	Brazil	45	4426	GeneActiv	Wrist	7
EPIMOV^p^ [65]	Observational	Brazil	42	1040	Actigraph GT3X	Waist	—
PACE-UP^q^ [66]	Interventional	United Kingdom	59	1023	Actigraph GT3X	Hip	7
INFORM^r^ [67]	Interventional	United Kingdom	—	956	Axivity AX3	Wrist	7
Effectiveness of physical activity intervention among government employees with metabolic syndrome [68]	Interventional	Malaysia	—	165	Lifecorder e-step	—	3
Effects of substituting sedentary behavior with light and moderate to vigorous physical activity on obesity indices in adults [69]	Abstract only	—	—	780	Actigraph GT3X	Waist	4
Whitehall [70]	Longitudinal	United Kingdom	66	445	Actigraph GT3X	—	7
RISC^s^ [71]	Observational	13 European countries	58	801	Actigraph AM7164	Waist	8
SCAPIS^t^ (pilot study) [72]	Observational	Sweden	—	661	Actigraph GT3x-BT	Hip	7
Identifying associations between sedentary time and cardiometabolic risk factors in working adults using objective and subjective measures: a cross-sectional analysis [73]	Observational	Japan	—	671	Omron Active Style Pro HJA 350-IT	—	—
Heredity and Phenotype Intervention Heart Study [74]	Interventional	United States	42.9	671	Actical	Hip	7
Canadian Nurse [75]	Observational	Canada	—	472	Actigraph GT3X	Right hip	9
Insulin resistance in Chileans of European and Indigenous descent: evidence for an ethnicity × environment interaction [76]	Observational	Chile	80.1	873	Actigraph ActiTrainer	Left hip	7
Rush Memory and Aging Project [77]	Longitudinal	United States	69.8	1556	Phillips Actical	Wrist	10
REGARDS^u^ [78]	Observational	United States	65	7873	Actical	Waist	7
PREDIMED^v^-plus [79]	Interventional	Spain	—	6000	GeneActiv	—	—
British Birth Cohort [80]	Observational	United Kingdom	61.1	4756	ActivPAL 3	Thigh	—
PURE^w^ [81]	Longitudinal	South Africa	50.6	189	Actiheart	Chest	7
DonorInsight [82]	Longitudinal	Holland	—	807	Actigraph GT3X or GT3X-BT	Waist	—
Light-intensity physical activity is associated with insulin resistance in elderly Japanese women independent of moderate to vigorous intensity physical activity [83]	Observational	Japan	27.6	807	Actimarker EW4800	—	28
Low levels of physical activity are associated with dysregulation of energy intake and fat mass gain over 1 year [84]	Longitudinal	United States	51.1	421	SenseWear Mini Armband	Arm	10
ERMA^x^ [85]	Observational	Finland	—	1393	Actigraph GT3X+ or wGT3X+	Waist	7
National Health Program [86]	Observational	Poland	47.9	1471	Actical	—	—
Objectively measured light-intensity lifestyle activity and sedentary time are independently associated with metabolic syndrome: a cross-sectional study of Japanese adults [87]	Observational	Japan	—	483	Omron HJA-350IT	Right hip	7
Objectively measured physical activity of Vietnamese adults with type 2 diabetes: opportunities to intervene [88]	Observational	Vietnam	76	120	Actigraph GT3X	Hip	5
Longitudinal assessment of bariatric surgery [89]	Longitudinal	United States	68.5	927	StepWatch 3	Ankle	7
Hisayama [90]	Longitudinal	Japan	29.9	1758	Omron Active Style Pro	—	—
MESA^y^ Sleep Study^d^ [91]	Observational	United States	43.9	2000	Phillips Actiwatch	Nondominant wrist	7
Stork Groruddalen Study [92]	Observational	Norway	—	759	SenseWear Armband Pro3	—	4
Inuit Health in Transition study [93]	Observational	Greenland	71.9	1497	ActiHeart	Chest	5
Jackson Heart Study [94]	Observational	United States	72.7	423	Actigraph 7164	Waist	1
Walking and leg circulation study (subsample) [95]	Observational	United States	48.9	460	Caltrac	—	7
VIBE^z^ [96]	Observational	United Kingdom	68.9	1182	GCDC X15-1c	Waist	7
AusDiab [97]	Observational	Australia	53.7	3352	Actigraph 7164 and ActivPAL 3	Waist and thigh	
European Prospective Investigation into Cancer and Nutrition–Norfolk [98]	Longitudinal	United Kingdom	52.7	2012	Actigraph GT1M or GT3X	Right hip	7
Relationship between metabolic syndrome, circadian treatment time, and blood pressure nondipping profile in essential hypertension [99]	Observational	Spain	53	1006	Mini-motion logger Ambulatory Monitoring	Wrist	2
Risk of new-onset diabetes: influence of class and treatment-time regimen of hypertension medications [100]	Abstract only	Spain	—	2012	—	—	—
Seasonal variation of fibrinogen in dipper and nondipper hypertensive patients [101]	Observational	Spain	48	508	Mini-motion logger Ambulatory Monitoring	Wrist	—
Osteoartheritus Initiative [102]	Abstract only	United States	—	969	—	—	—
Ryobi Health Survey [103]	Observational	Japan	59.8	691	Omron Active Style Pro	—	7
Rotterdam Study [104]	Observational	Netherlands	44	1116	GeneActiv	Wrist	—
Early activity in diabetes [105]	Interventional	United Kingdom	46	528	Actigraph GT1M	Waist	—
Child Health Checkpoint [106]	Observational	Australia	—	877	GeneActiv	Wrist	8
European Fans in Training [107]	Interventional	United Kingdom, Netherlands, Norway, and Portugal	48	1113	ActivPAL 3	Thigh	7
Examining Neighborhood Activity in Built Living Environments London [108]	Observational	United Kingdom	56.1	877	Actigraph GT3X+	Hip	7
The morning surge in blood pressure and heart rate is dependent on levels of physical activity after waking [109]	Observational	Ireland	61	420	Gaehwiler Electronics	Wrist	—
Prospective Rural Urban Epidemiology (subsample) [110]	Observational	South Africa	—	341	Actigraph GT3X+	—	4
Innovative Medicines Initiative Diabetes Research on Patient Stratification cohorts [111]	Observational	Denmark, Sweden, Netherlands, United Kingdom, and Finland	72	1355	Actigraph GT3X+	Nondominant wrist	10
Tromsø [112]	Observational	Norway	69.5	3653	Actigraph wGT3X-BT	Right hip	8
Baltimore Longitudinal Aging Study [113]	Longitudinal	United States	—	546	ActiHeart	Chest	7
Migration and Ethnicity on Diabetes In Malmö (MEDIM) [114]	Observational	Sweden	47.7	962	Actigraph GT1M	Waist	—
Oulu 45 [115]	Abstract only	Finland	59.6	570	Polar Electro	Wrist	—
Toyota Motor Corporation Physical Activity and Fitness [116]	Abstract only	—	71.8	756	—	—	—
Mitchelstown cohort [117]	Observational	Ireland	52.6	3344	ActivInsight GeneActiv	Wrist	7
Heijo-Kyo [118]	Observational	Japan	40	528	Respironics ActiWatch 2	Wrist	2
Gender differences in ambulatory blood pressure thresholds for defining hypertension based on cardiovascular outcome [119]	Abstract only	Spain	—	3344	—	Wrist	—
Associations between self-reported and objectively measured physical activity and overweight or obesity among adults in Kota Bharu and Penang, Malaysia [120]	Observational	Malaysia	—	490	Actigraph GT3X+	Waist	7
Interactive Diet and Activity Tracking in American Association of Retired People study [121]	Observational	United States	63.2	584	Actigraph GT3X	Waist	7
Genes Environment Diabetes and Obesity [122]	Observational	Chile	—	409	Actigraph GT1M	Right hip	7
Latin American Study of Nutrition and Health [123]	Observational	Argentina, Brazil, Chile, Colombia, Costa Rica, Ecuador, Peru, and Venezuela	38.3	2524	Actigraph GT3X	Right hip	7
Echocardiographic Study of Latinos [124]	Observational	United States	56.4	1206	Actical B1	Waist	—
Community of Mine [125]	Observational	United States	59	598	Actigraph GT3X+	Hip	14
Korean National Health and Nutrition Evaluation Survey^d^ [126]	Observational	South Korea	48.9	2197	Actigraph GT3X	Waist	7
Dynamics of Lifestyle and Neighborhood Community on Health Study^d^ [127]	Observational	Japan	58.3	440	Omron Active Style Pro	Left hip	14
Alberta Moving Beyond Breast Cancer [128]	Observational	Canada	56	1528	Actigraph GT3x and ActivPal 3	Waist and thigh	7
Lifestyle Biomarkers and atherosclerosis Study [129]	Observational	Sweden	—	658	Actigraph GT3X+	Waist	7
Brazilian Longitudinal Study of Adult Health [130]	Observational	Brazil	—	0	Actiwatch 2	Nondominant wrist	7
Effect of Reducing Sedentary behavior on Blood Pressure [131]	Interventional	United States	—	0	Actigraph GT3x and AvticPAL 3	Waist and thigh	9
National Social, Health, and Aging project [132]	Observational	United States	—	1023	—	—	—
Vitamin and Lifestyle Intervention for gestational diabetes mellitus prevention [133]	Interventional	Austria, Belgium, Denmark, Ireland, Italy, Netherlands, Poland, Spain, and United Kingdom	32	436	Actigraph GT1M, GT3x+, and Actitrainer	Right hip	3
Middle-aged Soweto Cohort [134]	Observational	South Africa	—	727	Actigraph GT3x and ActivPal 3	Right hip and thigh	7
Stand More at Work and Life [135]	Interventional	United Kingdom	44.7	756	ActivPal 3, Axivity AX3	Thigh, wrist	8
Chronotype of Patients with Type 2 Diabetes and Effect on Glycaemic Control [136]	Observational	United Kingdom	—	998	Geneactiv	Wrist	8
Physical activity and health in older women [137]	Observational	China	65	1105	Actigraph wGT3x-BT	Left waist	7
Objectively measured step cadence and walking patterns in a rural African setting: a cross-sectional analysis [138]	Observational	South Africa	35.1	236	Actigraph AM-7164-2.2	Waist	7

^a^Denotes that a variable was not retrieved for that data set.

^b^MetS: Metabolic Syndrome.

^c^EVIDENT: Effectiveness of Internet-based Depression Treatment.

^d^Denotes data sets that were identified as open access.

^e^OPACH: The Objective Physical Activity and Cardiovascular Health Study.

^f^CARDIA: The Coronary Artery Risk Development in Young Adults Study.

^g^PROPEL: The Promotion of Physical Activity through structured education with differing levels of ongoing support for those with prediabetes.

^h^HCHS: Hispanic Community Health Study.

^i^SOL: Study of Latinos.

^j^PATH: Population Assessment of Tobacco and Health.

^k^ALSPAC: Avon Longitudinal Study of Parents and Children.

^l^PREVIEW: Prevention of diabetes through lifestyle intervention and population studies in Europe and around the World.

^m^CHMS: Canadian Health Measures Survey.

^n^DPHACTO: Danish Physical Activity cohort with Objective Measures.

^o^MAPEC: Monitorización Ambulatoria para Predicción de Eventos Cardiovascular.

^p^EPIMOV: Epidemiology of Human Movement Study.

^q^PACE-UP: Pedometer and consultation evaluation–UP.

^r^INFORM: Information and Risk Modification Trial.

^s^RISC: Relationship between Insulin Sensitivity and cardiovascular disease.

^t^SCAPIS: Swedish Cardiopulmonary BioImage Study.

^u^REGARDS: Reasons for Geographic and Racial Differences in Strokes.

^v^PREDIMED: Primary Prevention of Cardiovascular disease with a Mediterranean diet.

^w^PURE: Prospective Urban Rural Epidemiology.

^x^ERMA: Estrogenic Regulation of Muscle Apoptosis.

^y^MESA: multiethnic study of atherosclerosis.

^z^VIBE: Vertical Impacts on Bone in the Elderly.

**Figure 2:**
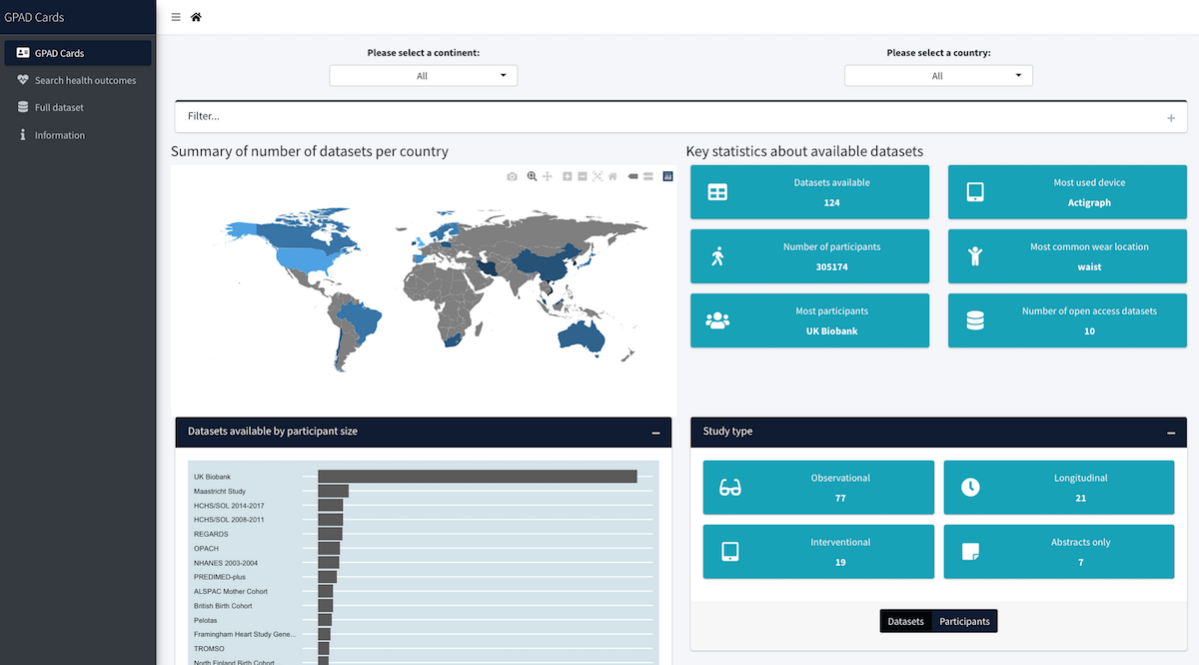
A screenshot of the home page of the Global Physical Activity Data set (GPAD) cards’ web-based data visualization tool.

**Figure 3:**
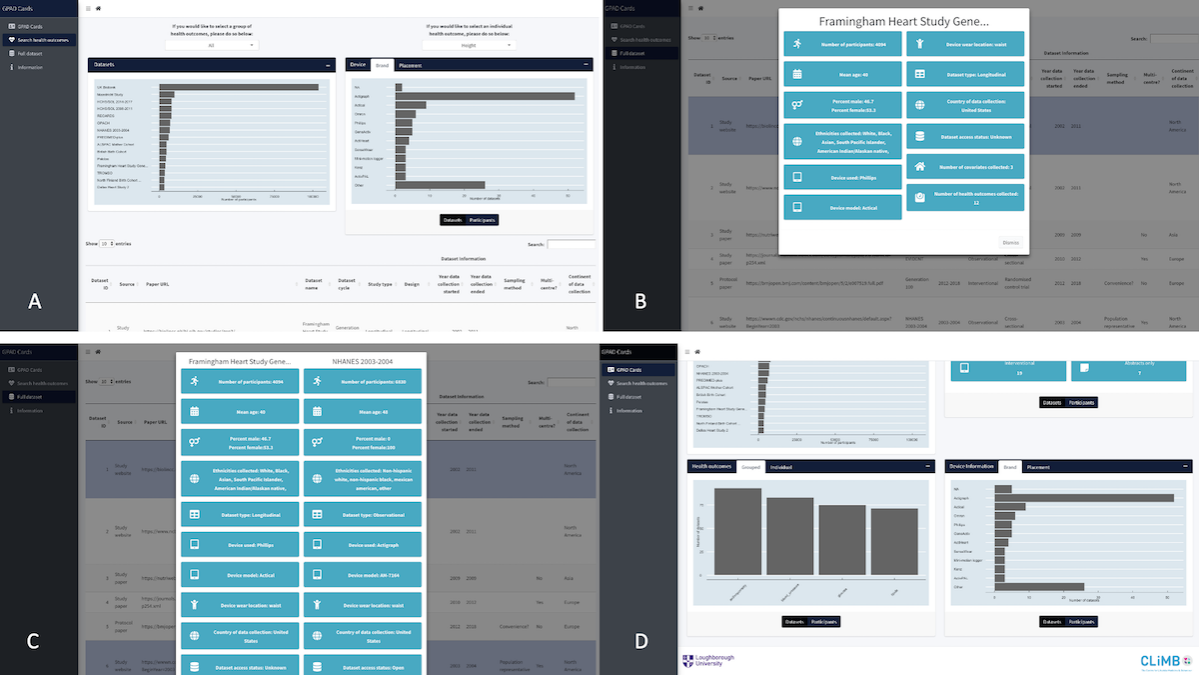
More detailed screenshots of the Global Physical Activity Data set (GPAD) catalogue tool. (A) The health markers screen that allows data sets shown to be filtered by the health markers they collect, (B) a visualization of a single data set within the tool, (C) a comparison of 2 data sets within the GPAD, and (D) the bottom of the home page showing grouped health markers and accelerometer information.

The GPAD catalogue, as shown in Figures 2 and 3, is a web-based tool developed to highlight the findings of this review. Several filters can be applied, including the continent or country of data collection, age, sex, ethnicity, and accelerometric device used. The complete catalog of data sets is also available on a separate page where a single data set can be selected, and a summary can be provided. Alternatively, 2 data sets can be selected and compared using a side-by-side summary of each. In addition, the users can select either a single health marker or a group of health markers, and a visual summary of all data sets that have collected the given health marker or markers is produced. The GPAD catalogue [20] is available on the web, and the source code can be found on GitHub [139].

### Narrative Summary

From the 122 unique data sets collected, 301,075 individual participants’ data were identified. Of the data sets where sex could be retrieved (111/122, 90.9%), 131,700 participants were female and 101,848 were male. The mean age of the participants was 54.1 (SD 12.35) years, with a range of 15 to 98 years, where mean age could be retrieved from the data set (110/122, 90.2%). Data sets were identified in 5 continents, with the majority being retrieved from Europe and North America (n=85 data sets) [5,21,22,24,25,28,31-36,39-45,48-50,52,55-58,60-63,66,67,70-72,​^74^,^75^,^77^-^80^,^82^,^84^-^86^,^89^,^91^-^96^,^98^-^102^,^104^,^105^,^107^-^109^,^111^-^115^,^117^,​^119^,^121^,^124^,^125^,^128^,^129^,^131^,^132^,^140^]. A wide range of health markers have been collected, with most studies measuring height (119/122, 97.5%), body mass (119/122, 97.5%), and blood pressure (82/122, 67.2%).

### Study Design and Data Access Status

Of the identified data sets, the majority (n=75) had an observational study design, 21 were longitudinal, and 19 were interventional. For 7 data sets, only an abstract could be found, and the study design could not be retrieved. The access status of the 36 data sets could be ascertained. Of these, 37 data sets were publicly available, 22 mentioned explicitly being available on request from the authors, and 5 reported that the data could not be shared.

### Countries of Data Collection

Seven studies were collected across multiple countries. Of these cross-country studies, 5 were collected solely in Europe; 1 was collected in European countries, Australia, and New Zealand; and 1 was collected across 8 South American countries. Most data sets were collected in the United States (n=28) and the United Kingdom (n=18). The United Kingdom had the largest volume of participant data (n=135,544), followed by the United States (n=67,040). Africa and South America had a smaller number of data sets collected (n=12 data sets combined). A total of 18 data sets were located in Asia; however, all had a relatively small mean number of participants per data set (mean 605; range 120-1758).

**Figure 4:**
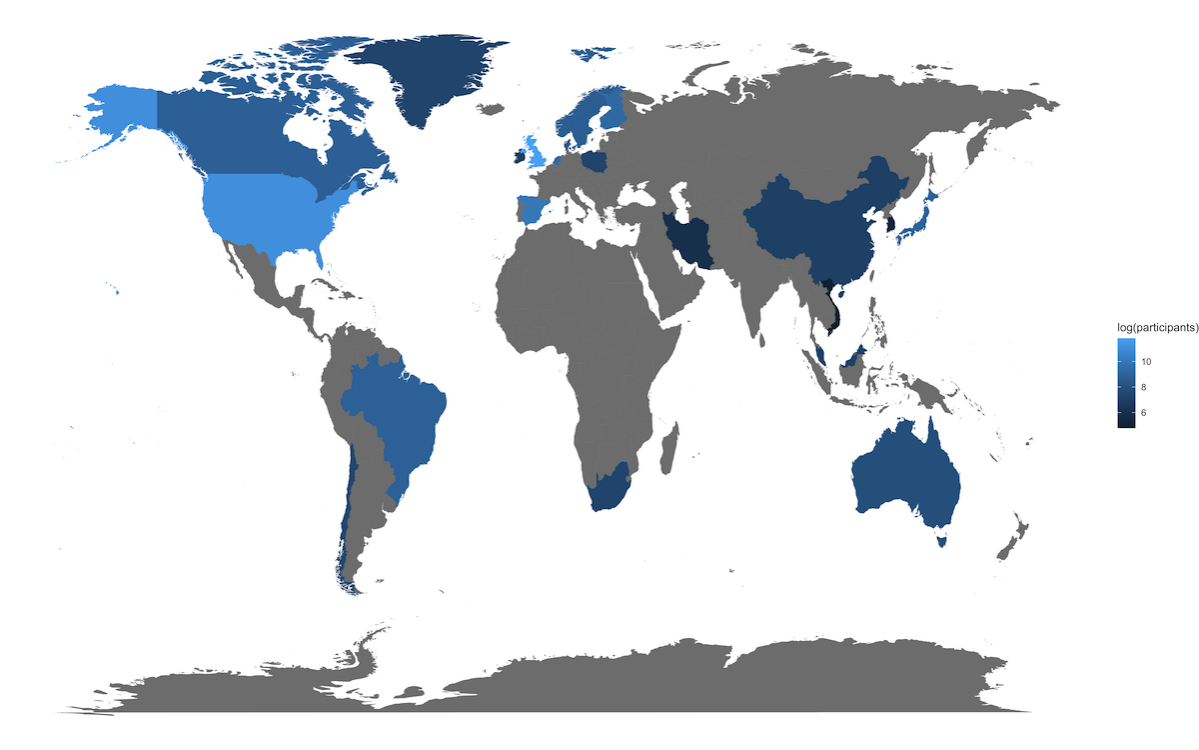
Choropleth world map showing countries where data sets have been collected and how many participants’ data have been collected in each country (log transformed).

A limited number of data sets (n=13) were identified within LMICs. These data sets were collected in Malaysia, Chile, Iran, South Africa, and Vietnam. They contained data on 4955 participants, accounting for 1.64% (4955/301,075) of the total number of identified participants. The average number of participants in data sets within these countries was 381. Figure 4 illustrates the countries in which data were collected, with a darker color representing fewer participants.

### Accelerometric Devices Used

Actigraphs and Actical devices were the most commonly used accelerometric devices (n=63). The most commonly used Actigraph model was GT3X (ActiGraph; n=38). Six wear locations were identified in the retrieved data sets. The 3 most common wear locations were the waist (n=63), followed by the wrist (n=22) and the thigh (n=11).

### Accelerometer Initialization and Processing

Only limited information could be retrieved on the methods surrounding device deployment and processing (raw data sampling frequency, choice of epoch, number of axes measured, and number of days of wear). For 29 data sets, it was not possible to determine the number of days an accelerometer was worn; for 73 data sets, it was not possible to determine the epoch used to analyze physical activity; for 69 data sets, the number of axes over which the device was initialized could not be retrieved; and for 94 data sets, the sampling rate used could not be retrieved. From what could be extracted, the most common number of days of wear was 7 (63/122, 51.6% of total data sets identified), whereas the most used epoch and raw data sampling frequency were 60 seconds (30/122, 24.6% of total data sets identified) and 30 Hz (11/122, 9%), respectively.

### Cardiometabolic Health Markers

**Figure 5:**
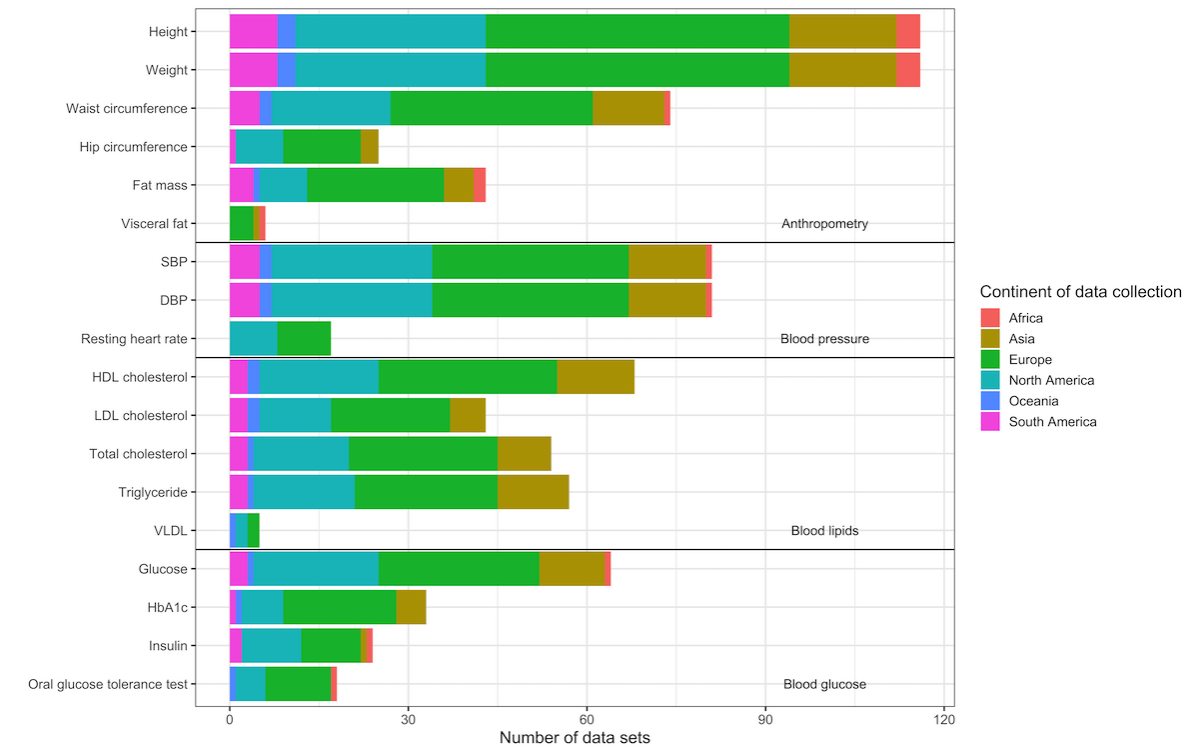
Number of data sets that collected each health outcome split by the continent of data collection. DBP: diastolic blood pressure; HDL: high-density lipoprotein; LDL: low-density lipoprotein; SBP: systolic blood pressure; VLDL: very low-density lipoprotein.

Height and body mass were the most reported health markers, with the data collected in 119 data sets (n=303,144 participants). Blood pressure (n=82 data sets and n=270,961 participants), waist circumference (n=75 data sets and n=253,683 participants), HDL cholesterol (n=68 data sets and n=239,113 participants), and blood glucose (n=65 data sets and n=225,185 participants) were the most collected cardiometabolic health markers. When health markers were broken down by the continent they were collected in, North America and Europe collected the widest range of health markers. Africa only reported studies that have measured more basic health markers (height, body mass, and blood pressure). Figure 5 shows the data sets which collected each health outcome split by the continent of data collection. Larger images of Figures 3-5 are provided in Multimedia Appendix 5.

### Grouped Health Markers

When health markers were grouped (based on the criteria defined in Multimedia Appendix 2), a total of 92 data sets measured anthropometry, 82 measured blood pressure, 75 measured blood glucose, and 70 measured blood lipids. When the collected grouped health markers were examined across continents, Africa only had data collected on anthropometry and blood pressure, whereas North America, Europe, Asia, South America, and Oceania had data sets that collected health markers across all 4 categories.

## Discussion

### Principal Findings

Accelerometer-measured physical activity has been collected alongside cardiometabolic health outcomes in many published data sets, which can be pooled to produce more heterogeneous data, providing greater statistical power. However, comprehensively identifying data sets to be included in pooled analysis is a time-consuming endeavor. The findings from this review informed the creation of the GPAD catalogue, a web-based resource to reduce the burden of identifying relevant data sets and allow researchers and other interested parties to explore and address important research questions regarding physical activity and cardiometabolic health in an efficient manner. The GPAD catalogue also includes 7 data sets from LMICs.

### Previous Research

A review by Wijndaele et al [19] of accelerometer-measured data sets identified Actigraphs as the most frequently used accelerometric device (39/76, 51% of identified data sets). This is similar to our review, with 41.8% (51/122) of the data sets using Actigraphs. Compared with the previous review by Wijndaele et al [19], this review benefits from the inclusion of an additional 29 data sets. Both reviews adopted a threshold of 400 participants as an eligibility criterion for the inclusion of data sets, but our review reduced this limit for LMICs (100 participants) to ensure there was data representation from across the world and from different ethnic and cultural groups. Our review also extends the work of Wijndaele et al [19] by extracting more variables from the data sets, which will facilitate future harmonization efforts. A more recent review and expert statement published in 2020 that collected thigh-worn accelerometer-measured physical activity included only data sets using observational study designs [141]. In contrast, all intervention or observational study designs were eligible for this review, resulting in more data sets being available for inclusion.

### The GPAD Catalogue

Although other reviews have aimed to identify data sets that have measured physical activity using an accelerometer [19,141], to our knowledge, this is the first study to create an interactive web-based catalog that can inform future harmonization and data pooling processes. The GPAD catalogue serves a similar purpose to the Maelstrom Catalogue, with a greater emphasis on accelerometer-measured physical activity. The Maelstrom Catalogue is a data discovery tool that allows users to identify data sets that may help to answer novel research questions as well as provide information on the data sets, which may ease the data harmonization process [142]. The GPAD catalogue also implemented elements of the Global Observatory of Physical Activity country cards by using visualizations to communicate information in an understandable and comparable manner to interested parties and organizations.

The concept of a repository for data sets with similar variables is not new and exists in several other research fields. For example, the database of Genotypes and Phenotypes is a database that archives and distributes the data and results from studies that have investigated the genotype and phenotype in humans, allowing users to identify data sets that may be of interest to them [143]. By providing this information in a central resource, the database of Genotypes and Phenotypes has the same aim as the GPAD catalogue, which is to increase collaboration, facilitate the processes of research, and reduce researcher burden. However, the GPAD catalogue does not aim to hold or provide access to individual study data; rather, it provides a summary of what is available to researchers if they were to collaborate with the appropriate data set owners. It is hoped that by taking a continual and collaborative approach to the development of the GPAD catalogue, frequent updates and adaptations can be made so that the resource remains relevant to the inclusion of new data sets over time.

### Accelerometer Device Use, Wear Location, and Reporting

Actigraph, followed by Actical, were the most used devices both regarding the number of data sets they were used in as well as the number of participants who wore them. The waist was the most used wear location (n=63), followed by the wrist (n=21) and the thigh (n=11). The dominance of the waist as a wear location is likely because of its extensive use in the early years of measuring physical activity using accelerometric devices. However, evidence from previous waist-worn data sets highlights that only approximately 25% of participants provided the requested 7 days of accelerometer wear data [144]. Wrist-worn devices have been shown to be more acceptable and lead to greater participant compliance [6,144]. In addition, advances in analytic techniques allowing posture to be assessed from the devices worn at the thigh means that both these wear locations are likely to increase in popularity over the coming years [145]. These findings highlight that the insights offered by a tool such as the GPAD catalogue can be used by researchers both for secondary data analysis and informing planned primary data collection.

A notable finding of this review was the lack of reporting of accelerometer deployment and analysis variables, consistent with a review by Montoye et al [146]. They found that, overall, the reporting of accelerometer variables was poor, with only marginal increases in reporting over time. Our findings corroborate this and highlight that future research should aim to report the key and detailed characteristics of the locations where accelerometers were worn alongside the analytic decisions taken to ease future pooling and harmonization efforts. Montoye et al [146] provided several key items that should be reported when an accelerometer is included in a study.

### Health Markers

Height and body mass were the most frequently collected health markers, followed by blood pressure. As evidence supports the role of both BMI and blood pressure as potential risk factors for cardiovascular disease [147,148], this is not unexpected. Another trend from this review is that health markers that are easier to assess (require less expensive equipment or are less time-consuming) were more commonly collected than health markers that require more specialists or expensive equipment. When health markers were grouped into larger categories (defined in Multimedia Appendix 2), anthropometry was the most frequently collected data. LMICs collected anthropometry (11/11, 100% data sets) and blood pressure (6/11, 55% data sets) data frequently; however, blood lipids and blood glucose were measured less frequently (5/11, 45% and 6/11, 55% data sets, respectively).

### Geographic Distribution of Data

There was a notable lack of data sets collected in LMICs that met the inclusion criteria for this study, highlighting the need for more relevant data sets. The lack of data sets in these countries could also be explained by the extensive knowledge, skills, and funding required to conduct cardiometabolic health assessments, on a large number of participants, as well as how to effectively initialize, deploy, and analyze accelerometers and their data. Furthermore, the financial burden of purchasing the necessary number of accelerometric devices to collect a large data set poses additional challenges for LMICs. Therefore, the GPAD catalogue resource may prove particularly useful to researchers in such regions to answer questions using the existing data.

### Access Status

The data access status could not be determined for 69.7% (85/122) of data sets. This highlights the need for better reporting of the access status of the data used within publications, which would allow researchers aiming to conduct secondary analysis or data harmonization a greater level of understanding of how to seek access to the data sets. Notably, over time, the number of data sets reporting their access status has increased. Between 2000 and 2010, only 11 data sets reported their access status, and between 2011 and 2021, a total of 17 data sets reported their access status. This improved reporting shows that journals requiring data set access statements may ease the harmonization process for future researchers. However, a recent review found that only 14% of papers that included a data access statement responded to a request to share data, and of these, only 6.8% of the authors provided the requested data, indicating that a data access statement may not be sufficient to ensure data sharing [149].

### Strengths and Limitations

A key strength of this review was the assistance from an information specialist in devising the search strategy. This ensured that the systematic search for eligible data sets was as robust and complete as possible, increasing the probability that all available papers and data sets were identified. The large number of studies returned from the initial search required the eligibility criteria to be refined during the review process, ultimately resulting in a more focused review. By making the results available interactively on the web in the GPAD catalogue, this review allows the findings to be disseminated to more potential users. The extraction of variables from each data set is more comprehensive than that in previous reviews [19], allowing those seeking to harmonize findings from across data sets to have access to more information. By adopting a lower participant number inclusion criteria for LMICs, 7 data sets from countries such as Vietnam, Chile, and Iran were included, which otherwise would not have been the case. The inclusion of these data sets into a resource such as the GPAD catalogue is important for the integrity and representativeness of the resource. This is also important because it means that data from a wide range of ethnic and cultural groups can be included in future studies resulting from the GPAD and because physical activity patterns will differ across geographic regions of the world. Moreover, this highlights how important it is for future reviews to include methodological decisions from the outset that will allow such data sets to be part of data catalogs.

This review has some limitations that should be considered. Data sets were only identified through published reports identified by our systematic searches; it is possible that some data sets may have been collected but not yet published. This was mitigated by searching for a wide range of sources, including gray literature and trial registries. Furthermore, it is possible that data sets may have collected health markers that are yet to be published and therefore may not be included within the GPAD catalogue. It was also hoped that the methodology related to how each health marker was collected could be recorded. However, these data were poorly reported in most data sets and therefore were not discussed in this review. What data could be extracted has been made available in the GPAD catalogue data set. Although we aimed to extract key variables from each data set related to markers of cardiometabolic health and accelerometer-measured physical activity, we appreciate that certain variables that researchers may find useful are omitted from the review. We welcome collaborators to help add these variables to future iterations of the resource.

### Future Developments and Implications

We plan to update the GPAD catalogue resource periodically (at least once per year) to include new data sets as they become publicly available. Furthermore, on the release of the resource, an email will be sent to the primary investigators on each data set to make them aware of the resource and to encourage them to inform us if their data set contains variables currently omitted from the resource.

For example, as the Prospective Physical Activity, Sitting, and Sleep consortium grows [150], a greater number of thigh worn accelerometry data sets with cardiometabolic health information will become available in the future. Additional health markers, such as mental health and mortality outcomes, will also be added to the resource over time. We hope that this will further increase the usefulness of the resource and the ability of the GPAD catalogue to assist in the development of harmonized analyses on a broader range of research questions related to physical activity and health.

### Conclusions

This review represents the most comprehensive analysis of its kind conducted to date, with 122 data sets identified that have quantified physical activity using an accelerometer and assessed cardiometabolic health markers. We have shown that data sets exist in all 5 inhabited continents of the world that have used a wide range of devices to measure physical activity. Future efforts to collect larger data sets with more comprehensive health markers are required, particularly in LMICs. The GPAD catalogue was created to allow important questions about physical activity and cardiometabolic health to be answered in an efficient manner and to ultimately produce evidence that will reduce the likelihood that adults die from diseases related to physical inactivity.

## Appendix

Multimedia Appendix 1 Full search strategy.

Multimedia Appendix 2 Logical statements used to generate grouped health outcomes from extracted health outcomes.

Multimedia Appendix 3 Paper extraction table.

Multimedia Appendix 4 Data set extraction table.

Multimedia Appendix 5 Additional images of the Global Physical Activity Data set catalog.

## Abbreviations

GPAD: Global Physical Activity Data set
HDL: high-density lipoprotein
LMICs: low- and middle-income countries
NHANES: National Health and Nutrition Examination Survey
PRISMA-ScR: Preferred Reporting Items for Systematic Reviews and Meta-Analyses extension for Scoping Reviews

## Data Availability

The data sets generated or analyzed during this study are available in Multimedia Appendices 3 and 4 as well as on GitHub [139] and on the Global Physical Activity Data set catalogue Shiny app [20].
