# Supplementary material for: Accelerometer-Measured Physical Activity Data Sets (Global Physical Activity Data Set Catalogue) That Include Markers of Cardiometabolic Health: Systematic Scoping Review
Source: J Med Internet Res. 2023 Jul 19;25:e45599. doi: 10.2196/45599 (PMC10398367; doi:10.2196/45599)
Supplement: Multimedia Appendix 1 [file jmir_v25i1e45599_app1.docx]

**SOURCES SEARCHED FOR WAISTWORN ACCELEROMETER REVIEW – Feb 2021**

**Database: Ovid MEDLINE(R) and In-Process & Other Non-Indexed Citations <1946 to February 01, 2021>**Search date: Feb 2^nd^ 2021

1 (Exercis* or "step count" or "stepcount" or "step-count" or "stair adj2 use" or walk* or run* or "screen time" or "screentime" or "screen-time" or stand* or sit* or (physical* activ* adj5 sleep*)).ti,ab. (3782829)

2 ((physical* adj3 (activ* or fit* or inactiv*or therap*)) or (MVPA or PADL or SB or PA)).ti,ab. (223212)

3 exp Exercise/ or exp physical exertion/ or exp physical fitness/ or exp Sedentary Behavior/ (272715)

4 1 or 2 or 3 (4004206)

5 (Acceleromet* or actigraph* or Actimet* or activity track* or activity monitor*).ti,ab. or exp Accelerometry/ (27806)

6 Epidemiologic studies/ or exp case control studies/ or exp cohort studies/ or Case control.tw. (2350779)

7 ((cohort adj (study or studies)) or Cohort analy$ or (Follow up adj (study or studies)) or (observational adj (study or studies))).tw. (378291)

8 (Longitudinal or Retrospective or Cross sectional).tw. or Cross-sectional studies/ (1245847)

9 randomized controlled trial.pt. or randomized.mp. or placebo.mp. (932399)

10 6 or 7 or 8 or 9 (3822953)

11 (Geneactiv or Actiheart or ActivPAL or Axivity).ti,ab. (631)

12 (Actigraph or Actical).ti,ab. (2989)

13 5 or 11 or 12 (27943)

14 4 and 10 and 13 (7691)

15 Adult/ (5092240)

16 exp Child/ (1940693)

17 exp Infant/ (1154675)

18 16 or 17 (2512417)

19 18 not 15 (1744417)

20 14 not 19 (5933)

21 exp Animals/ (23754895)

22 exp Humans/ (18975263)

23 21 not 22 (4779632)

24 20 not 23 (5887)

**Database: Embase <1974 to 2021 February 01>**Search date: 2^nd^ February 2021

--------------------------------------------------------------------------------

1 (Exercis* or "step count" or "stepcount" or "step-count" or "stair adj2 use" or walk* or run* or "screen time" or "screentime" or "screen-time" or stand* or sit* or (physical* active* adj5 sleep*)).ti,ab. (5012418)

2 ((physical* adj3 (activ* or fit* or inactiv*or therap*)) or (MVPA or PADL or SB or PA)).ti,ab. (310552)

3 exp Exercise/ or exp fitness/ or exp Sedentary Lifestyle/ (389907)

4 1 or 2 or 3 (5312776)

5 (Acceleromet* or actigraph* or Actimet* or activity track* or activity monitor*).ti,ab. or exp Accelerometry/ or exp Accelerometer/ (39708)

6 Clinical study/ or Case control study/ or Family study/ or Longitudinal study/ or Retrospective study/ or Prospective study/ (2070665)

7 Cohort analysis/ (665472)

8 (Cohort adj (study or studies)).mp. or (Case control adj (study or studies)).tw. or (follow up adj (study or studies)).tw. or (observational adj (study or studies)).tw. or (epidemiologic$ adj (study or studies)).tw. or (cross sectional adj (study or studies)).tw. (1029543)

9 random:.tw. or placebo:.mp. or double-blind:.tw. (1889740)

10 6 or 7 or 8 or 9 (4549863)

11 (Geneactiv or Actiheart or ActivPAL or Axivity).ti,ab. (858)

12 (Actigraph or Actical).ti,ab. (4651)

13 5 or 11 or 12 (39933)

14 4 and 10 and 13 (8725)

15 Adult/ (7564903)

16 exp Child/ (2698200)

17 exp Infant/ (1011322)

18 16 or 17 (2698200)

19 18 not 15 (1990545)

20 14 not 19 (6962)

21 exp Animals/ (26707295)

22 exp Humans/ (21950226)

23 21 not 22 (4757069)

24 20 not 23 (6882)

**CENTRAL**Search date: 2^nd^ February 2021

#1 (Exercis* or "step count" or "stepcount" or "step-count" or "stair NEAR/2 use" or walk* or run* or "screen time" or "screentime" or "screen-time" or stand* or sit*):ti,ab,kw (413326)

#2 (physical* NEAR/3 (activ* or fit* or inactiv*or therap*)) or (MVPA or PADL or SB or PA):ti,ab,kw (75872)

#3 ("physical activity" NEAR/5 sleep*" or "physical activities" NEAR/5 sleep*"):ti,ab,kw (930104)

#4 MeSH descriptor: [Exercise] explode all trees (24831)

#5 MeSH descriptor: [Physical Exertion] explode all trees (3863)

#6 MeSH descriptor: [Physical Fitness] explode all trees (3329)

#7 MeSH descriptor: [Sedentary Behavior] explode all trees (1129)

#8 #1 or #2 or #4 or #5 or #6 or #7 (447637)

#9 (Acceleromet* or actigraph* or Actimet* or "activity tracker" or "activity trackers" or "activity tracking" or "activity monitor" or "activity monitors" or "activity monitoring"):ti,ab,kw (7582)

#10 MeSH descriptor: [Accelerometry] explode all trees (947)

#11 (Geneactiv or Actiheart or ActivPAL or Axivity):ti,ab,kw (306)

#12 (Actigraph or Actical):ti,ab,kw (997)

#13 #9 or #10 or #11 or #12 (7665)

#14 #8 and #13 (5810)

#15 MeSH descriptor: [Adult] explode all trees (465246)

#16 MeSH descriptor: [Child] explode all trees (56347)

#17 MeSH descriptor: [Infant] explode all trees (32245)

#18 #16 or #17 (74214)

#19 #18 not #15 (53801)

#20 #14 not #19 (5380)

#21 MeSH descriptor: [Animals] explode all trees (595828)

#22 MeSH descriptor: [Humans] explode all trees (595767)

#23 #21 not #22 (61)

#24 #20 not #23 (5380)

Limit to trials = (5371)

**OPEN GREY**

Search date: 2^nd^ Feb 2021

acceleromet* and physical* = 66 (24 relevant records included)

acceleromet* and exercis* = 29 (16 relevant records included)

Geneactiv or Actiheart or ActivPAL or Axivity or Actigraph or Actical = 0 (0 relevant records included)

acceleromet* and sedentary = 13 (2 relevant records include)

acceleromet* and inactiv* = 8 (4 relevant records included)

acceleromet* and activ* = 75 (37 relevant records included)

acceleromet* and step count* or step-count or stepcount = 1 (1 relevant record included)

acceleromet* and PA = 15 (7 relevant records included)

acceleromet* and MVPA = 7 (2 relevant records included)

acceleromet* and PADL = 0 (0 relevant records included)

acceleromet* and SB = 0 (0 relevant records included)

**Total: 93 ; after internal duplicates removed 57**

**CT.gov**

Search date: 2^nd^ Feb 2021

Accelerometer and Interventional Studies and (sedentary or inactive) and (Adult or Older Adult) = 134

Accelerometer and Observational Studies and (sedentary or inactive) and (Adult or Older Adult) = 14

Accelerometer and Observational Studies and (exercise or physical activities) and (Adult or Older Adult) = 110

Accelerometer and Interventional Studies and (exercise or physical activities) and (Adult or Older Adult) = 723

Accelerometry and Interventional Studies and (exercise or physical activities) and (Adult or Older Adult) = 189

Accelerometry and Observational Studies and (exercise or physical activities) and (Adult or Older Adult) = 40

Accelerometry and Observational Studies and (sedentary or inactive) and (Adult or Older Adult) =7

Accelerometry and Interventional Studies and (sedentary or inactive) and (Adult or Older Adult) =34

**Total: 1251 = after duplicates removed 1036**

**WHO ICTRP**Search date 2^nd^ Feb 2021 (only basic search functionality available)

acceleromet* and exercis* = 94 (synonyms also searched)

acceleromet* and sedentary * = 44 (synonyms also searched)

acceleromet* or actigraph* or actimet* or geneactiv* or actiheart or ActivPaL or Axivity or actical” = 675

**TOTAL 813; after duplicates removed 652**

**Conference Proceedings Citation Index – Science (Web of Science)**

Search date: 5^th^ February 2021^[[1]](#footnote-1)^

# 1 TI=(Exercis* or "step count" or "stepcount" or "step-count" or (stair NEAR/2 use) or walk* or run* or "screen time" or "screentime" or "screen-time" or stand* or sit* or (physical* activ* NEAR/5 sleep*) ) (188,239)

# 2 AB=(Exercis* or "step count" or "stepcount" or "step-count" or (stair NEAR/2 use) or walk* or run* or "screen time" or "screentime" or "screen-time" or stand* or sit* or (physical* activ* NEAR/5 sleep*) ) 1,045,454

# 3 TI =(physical* NEAR/3 activ*)OR TI=(physical* NEAR/3 fit*)OR TI=(physical NEAR/3 inactiv*)OR TI=(physical NEAR/3 therap*)OR TI=(MVPA or PADL or SB or PA) (14,341)

# 4 AB =(physical* NEAR/3 activ*)OR AB=(physical* NEAR/3 fit*)OR AB=(physical NEAR/3 inactiv*)OR AB=(physical NEAR/3 therap*)OR AB=(MVPA or PADL or SB or PA) (36,466)

# 5 #4 OR #3 OR #2 OR #1 (1,183,421)

# 6 TI= (Acceleromet* or actigraph* or Actimet* or activit* track* or activit* monitor*) (6,965)

# 7 AB= (Acceleromet* or actigraph* or Actimet* or activit* track* or activit* monitor*) (43,661)

# 8 TI= (Geneactiv or Actiheart or ActivPAL or Axivity or Actigraph or Actical) (85)

# 9 AB= (Geneactiv or Actiheart or ActivPAL or Axivity or Actigraph or Actical) (105)

# 10 #9 OR #8 OR #7 OR #6 (46,329)

# 11 #10 AND #5 (13,107)

# 12 TS= clinical trial* OR TS=research design OR TS=comparative stud* OR TS=evaluation stud* OR TS=controlled trial* OR TS=follow-up stud* OR TS=prospective stud* OR TS=random* OR TS=placebo* OR TS=(single blind*) OR TS=(double blind*) 720,694

# 13 #12 AND #11 (1,711 )

# 14 TI= (cohort NEAR/1 stud*) or TI= (Cohort analy$) or TI = (Follow up NEAR/1 stud*) or TI = (observational NEAR/1 stud*) (18,577)

# 15 AB = (cohort NEAR/1 stud*) or AB= (Cohort analy$) or AB= (Follow up NEAR/1 stud*) or AB = (observational NEAR/1 stud*) . (12,481)

# 16 TI= (case control*) or AB = (case control*) (98,783)

# 17 TI = (Longitudinal or Retrospective or Cross sectional) or AB =(Longitudinal or Retrospective or Cross sectional) 118,845

# 18 #17 OR #16 OR #15 OR #14 (238,738)

# 19 #18 AND #11 (514)

# 20 #19 OR #13 (2,080)

**SPORT DISCUS (via EBSCO)**

Search date: 8^th^ Feb 2021

S1 TI Exercis* or "step count" or "stepcount" or "step-count" or "stair use" or walk* or run* or "screen time" or "screentime" or "screen-time" or stand* or sit* (377,217)

S2 AB Exercis* or "step count" or "stepcount" or "step-count" or "stair use" or walk* or run* or "screen time" or "screentime" or "screen-time" or stand* or sit* (407,998)

S3 TI ((physical* n3 (activ* or fit* or inactiv*or therap*)) OR ("physical* activ*" n5 sleep*) (30,518)

S4 AB ((physical* n3 (activ* or fit* or inactiv*or therap*)) OR ("physical* activit*" n5 sleep*) (53,303)

S5 TI PA or MVPA or PADL or SB (5,057)

S6 AB PA or MVPA or PADL or SB (7,231)

S7 (MH "Exercise+") (140)

S8 (MH "Physical Activity") (3)

S9 (MH "Life Style, Sedentary+") (64)

S10 S1 OR S2 OR S3 OR S4 OR S5 OR S6 OR S7 OR S8 OR S9 (479,705)

S11 TI Acceleromet* or actigraph* or Actimet* or "activit* track*" or "activit* monitor*" (3,507)

S12 AB Acceleromet* or actigraph* or Actimet* or "activit* track*" or "activity monitor*" (6,284)

S13 (MH "Accelerometers") (4)

S14 S11 OR S12 OR S13 (6,543)

S15 TI Geneactiv or Actiheart or ActivPAL or Axivity or Actigraph or Actical (1,293)

S16 AB Geneactiv or Actiheart or ActivPAL or Axivity or Actigraph or Actical (1,311)

S17 S14 OR S15 OR S16 (6,645)

S18 S10 AND S17 (5,150)

S19 MH Clinical Trials+ (22,553)

S20 PT Clinical Trial (18,467)

S21 TX clinic* n1 trial* (18,357)

S22 TX ( (singl* n1 blind*) or (singl* n1 mask*) ) or ( (doubl* n1 blind*) or (doubl* n1 mask*) ) or ( (tripl* n1 blind*) or (tripl* n1 mask*) ) or ( (trebl* n1 blind*) or (trebl* n1 mask*) ) (9,007)

S23 TX randomi* control* trial* (23,087)

S24 MH "Random Assignment" (181)

S25 TX random* allocat* (2,069)

S26 TX placebo* (12,603)

S27 MH Placebos (12,590)

S28 MH Quantitative Studies (1,940)

S29 TX allocat* random* (2,069)

S30 S19 OR S20 OR S21 OR S22 OR S23 OR S24 OR S25 OR S26 OR S27 OR S28 OR S29 (45,280)

S31 S18 AND S30 (358)

S32 (MH "Prospective Studies+") OR (MH "Case Control Studies+") (181)

S33 (MH "Correlational Studies") OR (MH "Cross Sectional Studies") (115)

S34 (MH "Nonconcurrent Prospective Studies") (3)

S35 TX (cohort n1 (study or studies) (10,063)

S36 TX (observational n1 (study or studies)) (4,494)

S37 S32 OR S33 OR S34 OR S35 OR S36 (14,040)

S38 S18 AND S37 (185)

S39 S31 OR S38 (532)

**CINAHL PLUS (Via EBSCO)**

Search date: 10^th^ Feb 2021

**S**1 TI Exercis* or "step count" or "stepcount" or "step-count" or "stair use" or walk* or run* or "screen time" or "screentime" or "screen-time" or stand* or sit* (864,145)

S2 AB Exercis* or "step count" or "stepcount" or "step-count" or "stair use" or walk* or run* or "screen time" or "screentime" or "screen-time" or stand* or sit* (894,594)

S3 TI ((physical* n3 (activ* or fit* or inactiv*or therap*)) OR ("physical* activ*" n5 sleep*) (31,087)

S4 AB ((physical* n3 (activ* or fit* or inactiv*or therap*)) OR ("physical* activit*" n5 sleep*) (62,157)

S5 TI PA or MVPA or PADL or SB (8,442)

S6 AB PA or MVPA or PADL or SB (18,937)

S7 (MH "Exercise+") (117,592)

S8 (MH "Physical Activity") (42,945)

S9 (MH "Life Style, Sedentary+") (8,758)

S10 S1 OR S2 OR S3 OR S4 OR S5 OR S6 OR S7 OR S8 OR S9 (1,016,558)

S11 TI Acceleromet* or actigraph* or Actimet* or "activit* track*" or "activit* monitor*" (6,498)

S12 AB Acceleromet* or actigraph* or Actimet* or "activit* track*" or "activity monitor*" (10,690)

S13 (MH "Accelerometers") (2,459)

S14 S11 OR S12 OR S13 (11,834)

S15 TI Geneactiv or Actiheart or ActivPAL or Axivity or Actigraph or Actical (1,963)

S16 AB Geneactiv or Actiheart or ActivPAL or Axivity or Actigraph or Actical (2,000)

S17 S14 OR S15 OR S16 (11,925)

S18 S10 AND S17 (8,884)

S19 MH Clinical Trials+ (314,497)

S20 PT Clinical Trial (109.001)

S21 TX clinic* n1 trial* (297,375)

S22 TX ( (singl* n1 blind*) or (singl* n1 mask*) ) or ( (doubl* n1 blind*) or (doubl* n1 mask*) ) or ( (tripl* n1 blind*) or (tripl* n1 mask*) ) or ( (trebl* n1 blind*) or (trebl* n1 mask*) ) (1,176,221)

S23 TX randomi* control* trial* (218,503)

S24 MH "Random Assignment" (66,284)

S25 TX random* allocat* (13,392)

S26 TX placebo* (70,766)

S27 MH Placebos (13,131)

S28 MH Quantitative Studies (28,843)

S29 TX allocat* random* (13,392)

S30 S19 OR S20 OR S21 OR S22 OR S23 OR S24 OR S25 OR S26 OR S27 OR S28 OR S29 (1,557,603)

S31 S18 AND S30 (3,150)

S32 (MH "Prospective Studies+") OR (MH "Case Control Studies+") (532,920)

S33 (MH "Correlational Studies") OR (MH "Cross Sectional Studies") (223,522)

S34 (MH "Nonconcurrent Prospective Studies") (249)

S35 TX (cohort n1 (study or studies) (103,210)

S36 TX (observational n1 (study or studies)) (53,623)

S37 S32 OR S33 OR S34 OR S35 OR S36 (792,811)

S38 S18 AND S37 (2,835)

S39 S31 OR S38 (5,187)

S40 (MH “Adult+”) (1,872,146)

S41 (MH “Child+”) (689,101)

S42 S41 not S40 (524,021)

S43 S39 not S42 (3,998)

**PubMed Updated Search (1^st^ September 2022)**

| **1** | Exercis*[Title/Abstract] OR "step count"[Title/Abstract] OR "stepcount"[Title/Abstract] OR "step-count"[Title/Abstract] OR "stair adj2 use"[Title/Abstract] OR walk*[Title/Abstract] OR run*[Title/Abstract] OR "screen time"[Title/Abstract] OR "screentime"[Title/Abstract] OR "screen-time"[Title/Abstract] OR stand*[Title/Abstract] OR sit*[Title/Abstract] OR (physical* activ* adj5 sleep*[Title/Abstract]) | 2,062,069 |
| --- | --- | --- |
| **2** | ((physical* adj3 (activ* or fit* or inactiv*or therap*)) or (MVPA or PADL or SB or PA)) | 4,271,992 |
| **3** | Exercise or physical exertion or physical fitness or Sedentary[MeSH Major Topic] | 568,040 |
| **4** | #1 OR #2 OR #3 | 6,233,176 |
| **5** | ((Acceleromet*[Title/Abstract] OR actigraph*[Title/Abstract] OR Actimet*[Title/Abstract] OR activity track*[Title/Abstract] OR activity monitor*[Title/Abstract])) OR (accelerometry[MeSH Major Topic]) | 31,481 |
| **6** | (Epidemiologic studies or case control studies or cohort studies[MeSH Major Topic]) OR (case control[Title/Abstract]) | 3,008,142 |
| **7** | (cohort adj (study[Title/Abstract] OR studies[Title/Abstract])) OR Cohort analy*[Title/Abstract] OR Follow up adj (study[Title/Abstract] OR (observational adj (study[Title/Abstract] OR studies[Title/Abstract]))) | 7,900 |
| **8** | ((Longitudinal[Title/Abstract] OR Retrospective[Title/Abstract] OR Cross sectional[Title/Abstract])) OR (or Cross-sectional studies/[MeSH Major Topic])) OR (or Cross-sectional studies[MeSH Major Topic]) | 1,324,860 |
| **9** | randomized controlled trial or randomized or placebo[MeSH Major Topic] | 1,358,170 |
| **10** | #6 or #7 or #8 or #9 | 4,504,696 |
| **11** | Geneactiv[Title/Abstract] OR Actiheart[Title/Abstract] OR ActivPAL[Title/Abstract] OR Axivity[Title/Abstract] | 809 |
| **12** | actigraph[Title/Abstract] OR actical[Title/Abstract] | 3,489 |
| **13** | #5 or #11 or #12 | 31,674 |
| **14** | #4 and #10 and #13 | 10,001 |

Filters applied: human population, adult and date 01/02/2021 and 01/09/2022.

Once filters applied: 671 reports returned

1. [↑](#footnote-ref-1)
