# Supplementary material for: Accelerometer-Measured Physical Activity Data Sets (Global Physical Activity Data Set Catalogue) That Include Markers of Cardiometabolic Health: Systematic Scoping Review
Source: J Med Internet Res. 2023 Jul 19;25:e45599. doi: 10.2196/45599 (PMC10398367; doi:10.2196/45599)
Supplement: Multimedia Appendix 2 [file jmir_v25i1e45599_app2.docx]

**Logical statements used to create grouped health markers**

To group the health markers together a series of IF statements were designed. For anthropometry, both height and body mass as well as one other measure of body size was required due to the large number of studies that measured height and body mass. For all other grouped health markers, measurement of a single relevant health outcome was sufficient.

| Group of health markers | Logical statement used to generate grouping |
| --- | --- |
| Anthropometry | IF measured height AND body mass AND waist circumference OR hip circumference OR fat mass OR visceral fat  THEN marked as YES (1)  ELSE marked as NO (0) |
| Blood pressure | IF measured systolic blood pressure OR diastolic blood pressure  THEN marked as YES (1)  ELSE marked as NO (0) |
| Blood lipids | IF measured HDL cholesterol OR LDL cholesterol OR Triglycerides OR VLDL  THEN marked as YES (1)  ELSE marked as NO (0) |
| Blood glucose control | IF measured blood glucose OR insulin OR HbA1c OR oral glucose tolerance test  THEN marked as YES (1)  ELSE marked as NO (0) |
