# Supplementary material for: Accelerometer-Measured Physical Activity Data Sets (Global Physical Activity Data Set Catalogue) That Include Markers of Cardiometabolic Health: Systematic Scoping Review
Source: J Med Internet Res. 2023 Jul 19;25:e45599. doi: 10.2196/45599 (PMC10398367; doi:10.2196/45599)
Supplement: Multimedia Appendix 5 [file jmir_v25i1e45599_app5.docx]

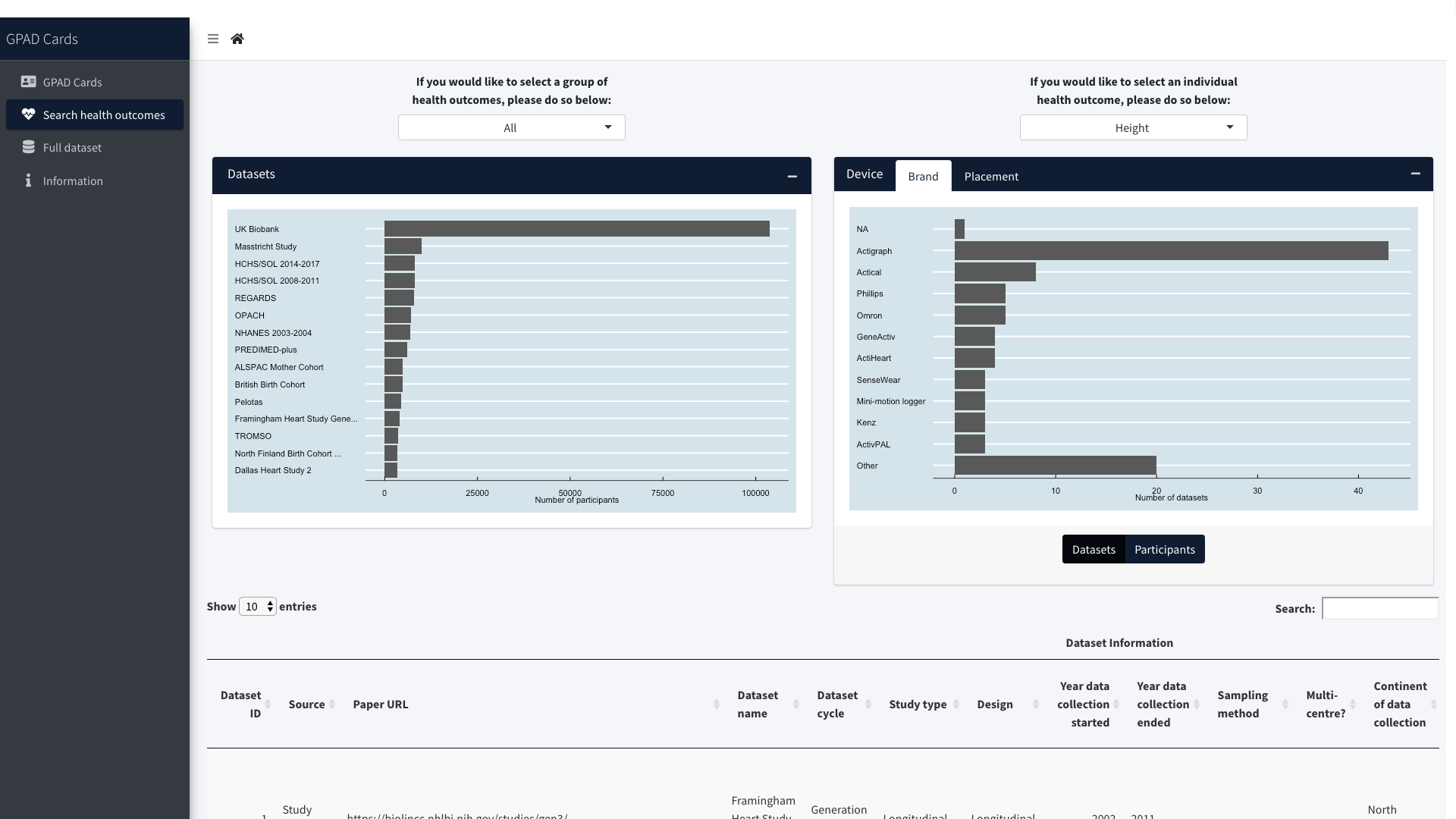


Figure 3a: the health markers screen that allows datasets shown to be filtered by the health markers they collect


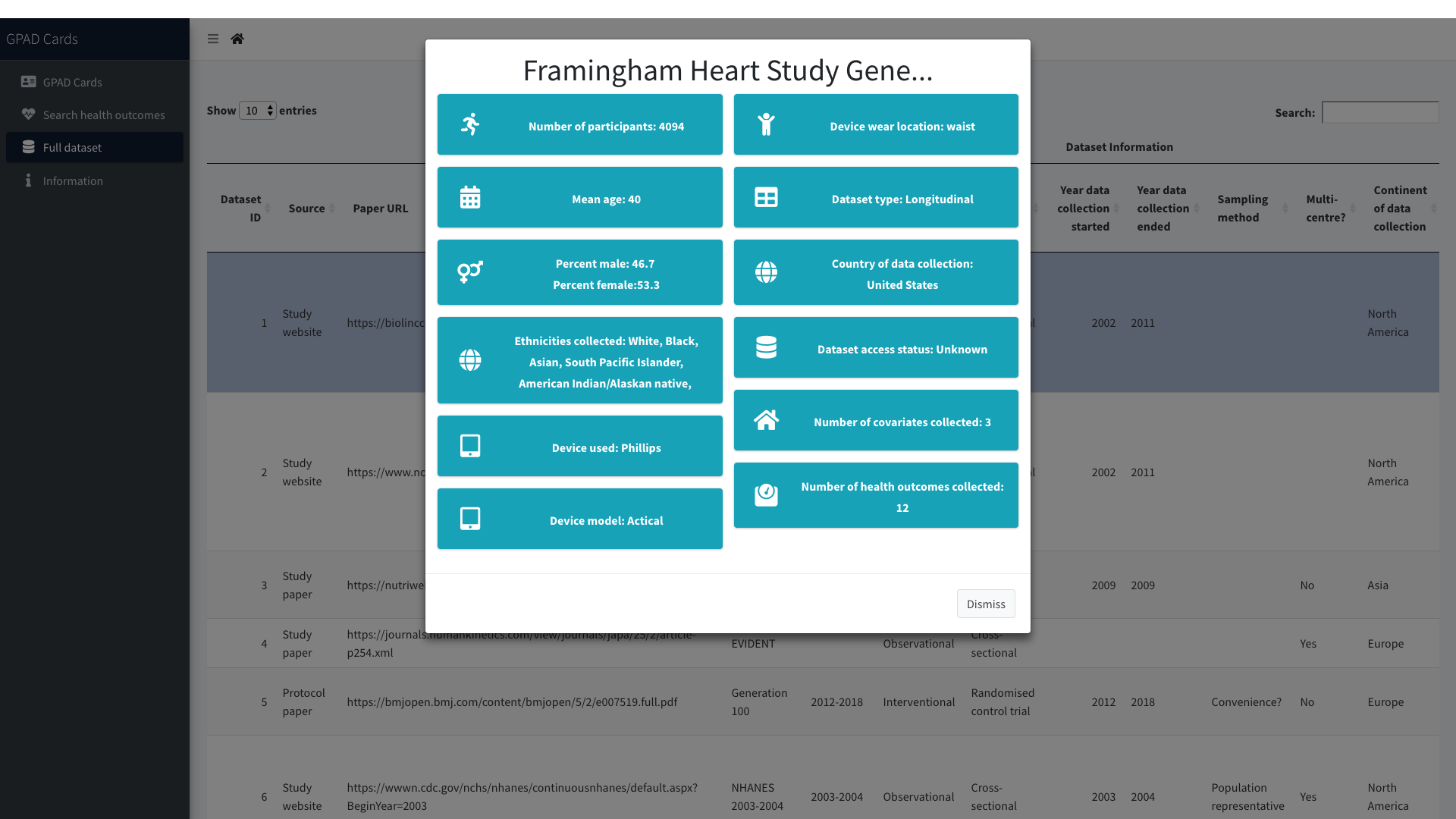


Figure 3b: a visualisation of a single dataset within the GPAD catalogue


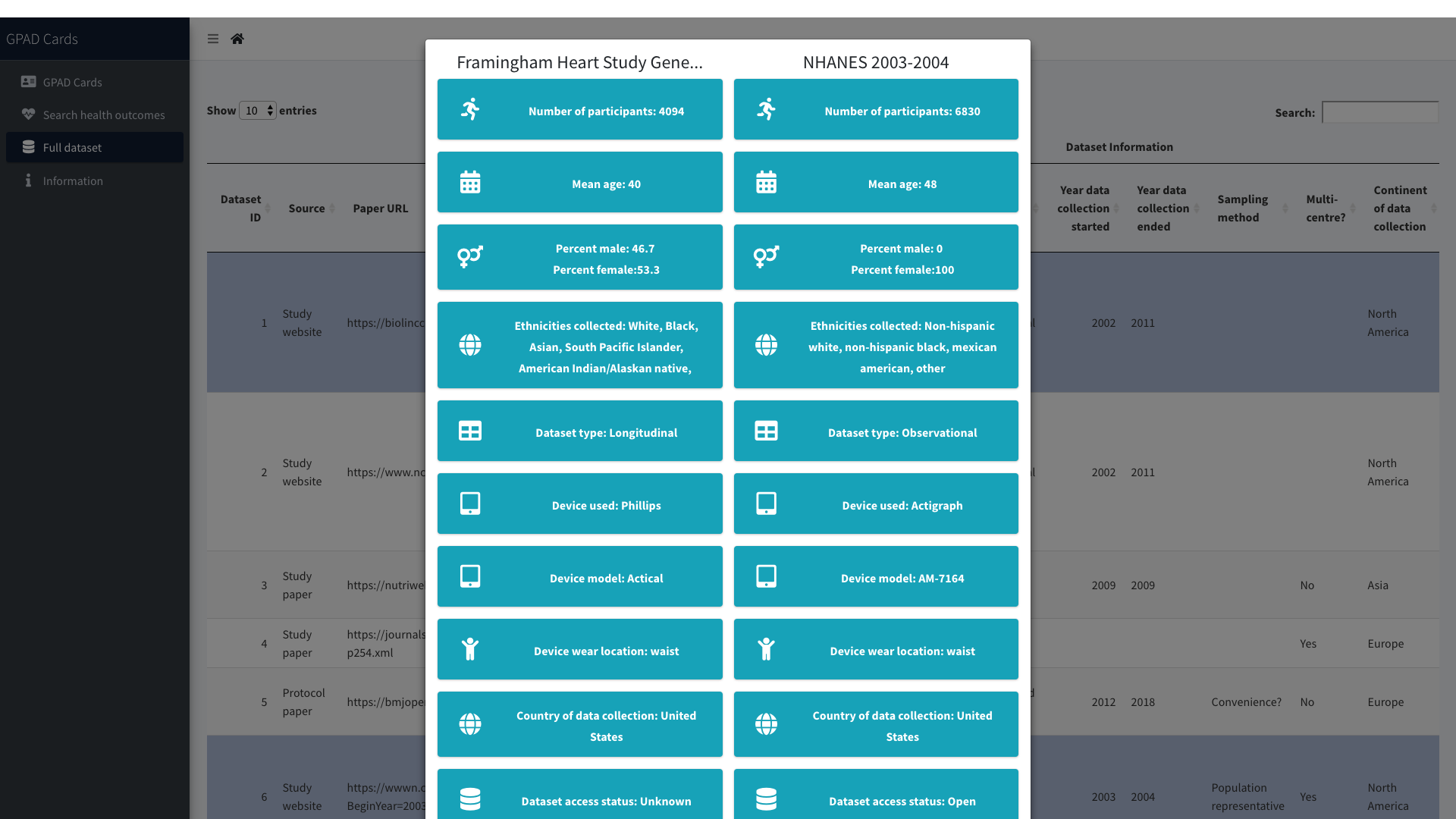


Figure 3c: a comparison of two datasets within the GPAD catalogue


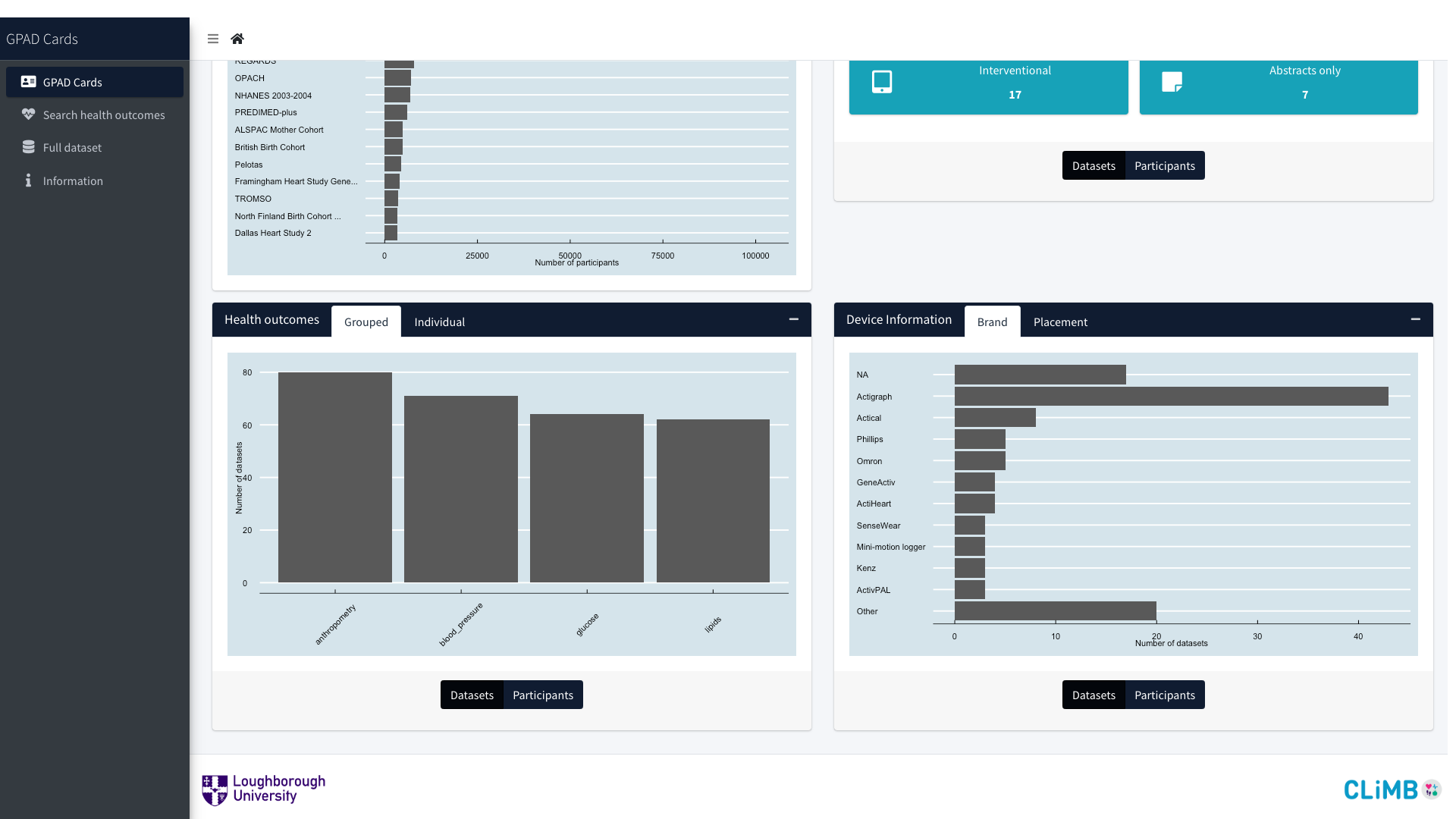


Figure 3d: the bottom of the home page showing grouped health markers and accelerometer information


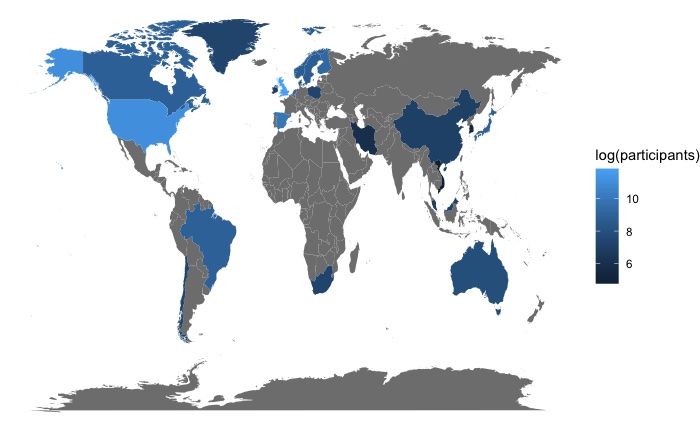


Figure 4. Choropleth World map showing countries where datasets have been collected and how many participants data has been collected in each country (log transformed).


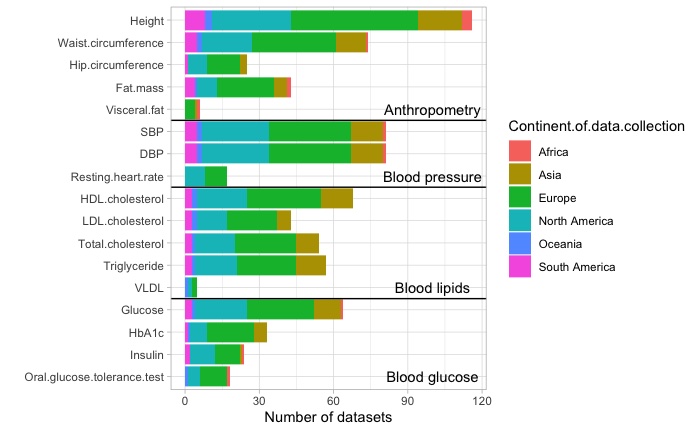


Figure 5. Number of datasets which collected each health outcome split by the continent of data collection.
